# Epidemiological characteristics, spatial clusters and monthly incidence prediction of hand, foot and mouth disease from 2017 to 2022 in Shanxi Province, China

**DOI:** 10.1017/S0950268823000389

**Published:** 2023-03-14

**Authors:** Yifei Ma, Shujun Xu, Ali Dong, Jianhua An, Yao Qin, Hui Yang, Hongmei Yu

**Affiliations:** 1 School of Public Health, Shanxi Medical University, Taiyuan, China; 2 Shanxi Center for Disease Control and Prevention, Taiyuan, China; 3 Supervision and Inspection Center of Health Commission of Shanxi Province, Taiyuan, China

**Keywords:** Epidemiological characteristics, Hand, foot and mouth disease, LSTM model, Monthly incidence prediction, SARIMA model, Spatial clusters

## Abstract

Hand, foot and mouth disease (HFMD) is a common infection in the world, and its epidemics result in heavy disease burdens. Over the past decade, HFMD has been widespread among children in China, with Shanxi Province being a severely affected northern province. Located in the temperate monsoon climate, Shanxi has a GDP of over 2.5 trillion yuan. It is important to have a comprehensive understanding of the basic features of HFMD in those areas that have similar meteorological and economic backgrounds to northern China. We aimed to investigate epidemiological characteristics, identify spatial clusters and predict monthly incidence of HFMD. All reported HFMD cases were obtained from the Shanxi Center for Disease Control and Prevention. Overall HFMD incidence showed a significant downward trend from 2017 to 2020, increasing again in 2021. Children aged < 5 years were primarily affected, with a high incidence of HFMD in male patients (relative risk: 1.316). The distribution showed a seasonal trend, with major peaks in June and July and secondary peaks in October and November with the exception of 2020. Other enteroviruses were the predominant causative agents of HFMD in most years. Areas with large numbers of HFMD cases were primarily in central Shanxi, and spatial clusters in 2017 and 2018 showed a positive global spatial correlation. Local spatial autocorrelation analysis showed that hot spots and secondary hot spots were concentrated in Jinzhong and Yangquan in 2018. Based on monthly incidence from September 2021 to August 2022, the mean absolute error (MAE), mean absolute percentage error (MAPE), and root mean square error (RMSE) of the long short-term memory (LSTM) and seasonal autoregressive integrated moving average (SARIMA) models were 386.58 *vs.* 838.25, 2.25 *vs.* 3.08, and 461.96 *vs.* 963.13, respectively, indicating that the predictive accuracy of LSTM was better than that of SARIMA. The LSTM model may be useful in predicting monthly incidences of HFMD, which may provide early warnings of HFMD epidemics.

## Introduction

Hand, foot and mouth disease (HFMD) is an acute infectious disease caused by enterovirus 71 (EV71), coxsackievirus A16 (CVA16) and other enteroviruses. As is well known, EV71 and CVA16 are the most common aetiological agents causing HFMD epidemics, but several studies have shown that other enteroviruses (non-EV71 and non-CVA16 enteroviruses), such as CVA6 and CVA10, appear to be on the rise since 2008 [1, 2]. Although approximately 30–90% of infections may be asymptomatic, some may result in severe manifestations such as myocarditis, neurological complications, and pulmonary oedema, which may eventually lead to death [3, 4]. HFMD has caused widespread social concern, especially in Asia and the Pacific Rim, such as China [5], Singapore [6], and Japan [7]. In mainland China, HFMD was first detected and reported in Shanghai in 1981, followed by large-scale epidemics in Shandong and Anhui provinces in 2007 and 2008 [8, 9]. According to the statutorily notifiable infectious disease epidemic report in July 2022, influenza, HFMD, and other infectious diarrhoeal diseases ranked the top three in the number of reported cases of Class C infectious diseases. Experts from the Chinese Center for Disease Control and Prevention (CCDC) have estimated that the transmission coefficient of HFMD is as high as 6.5, approximately three times that of early COVID-19, indicating the severity of HFMD as a public health hazard in China [10].

Comprehensive descriptions of the epidemiological characteristics and spatial clusters of infectious diseases, particularly at the provincial level, facilitate the implementation of targeted public health measures. In terms of epidemiological characteristics, researchers have investigated the epidemiology of HFMD in some areas of China, including Jiangsu Province [11], Shandong Province [12], and Qinghai Province [13]. Spatial autocorrelation analysis has recently been widely used in disease prevention and control, and researchers have applied this analytical method to explore the geographical distribution patterns of infectious diseases, including dengue fever [14], tuberculosis [15], as well as HFMD [16, 17]. Shanxi, located in northern China (34°58′-40°72′N, 110°25′-114°55′E), has a population size of 34.91 million and a GDP of over 2.5 trillion yuan in 2022. This province belongs to the temperate monsoon climate, characterised by hot, humid summers and cold, dry winters, which is conducive to the spread of HFMD [18, 19]. There is therefore a need to systematically understand the epidemiological and spatial distribution of HFMD in areas that are similar to northern China.

In recent years, the incidence of HFMD in Shanxi has been at the forefront of notifiable infectious diseases [20]. Although an inactivated monovalent EV71 vaccine was launched in 2016, HFMD remains a considerable public health challenge due to the vaccine being highly efficient against EV71-associated infection, but not against other aetiologies [21]. Therefore, establishing accurate prediction models is critical in estimating the trends of HFMD, which may strengthen prevention and control measures against epidemic. Early warning models are regarded as important tools for forecasting the occurrence of infectious diseases, among which the seasonal autoregressive integrated moving average (SARIMA) and long short-term memory (LSTM) models are particularly popular [22, 23]. Concerning specified time series, the SARIMA model is one of the optimal linear models that considers seasonality, periodicity, and long-term trends of the data. The LSTM network is a deep learning method that has been widely used for video classification, speech recognition, and disease prediction [24, 25]. LSTM can alleviate the problem of gradient disappearance or gradient explosion that occurs in traditional recurrent neural networks (RNN) or nonlinear autoregressive neural networks, which otherwise struggle to build long-term dependency structures in time-series. At present, LSTM model has been successfully applied to incidence prediction of Class C infectious diseases with slower transmission rate and lower prevalence and pathogenicity, such as influenza and mumps [26, 27]. Therefore, the use of LSTM model to forecast the incidence of HFMD, which is also a Class C infectious disease, is considered to be a beneficial exploration. In this study, we constructed an LSTM network, motivated by the high burden of HFMD in Shanxi, and compared its predictive accuracy with the SARIMA method to find the proper time-series modelling technique.

To develop appropriate provincial public health precautions, a comprehensive investigation of the fundamental characteristics of HFMD is needed. Our aims were to characterise the epidemiology of HFMD, explore global and local spatial autocorrelations, and build accurate prediction models to estimate the monthly incidence of HFMD in Shanxi. Our findings can provide beneficial reference for the prevention and control of HFMD in regions worldwide with similar meteorological and economic backgrounds to northern China.

## Methods

### Data collection

The monthly surveillance data of HFMD in Shanxi Province from 2017–2022 were obtained from 110 sentinel hospitals in 11 prefecture-level cities, providing a reasonably representative sample of HFMD cases during the study period. The responsible reporter should fill in the Infectious Disease Report Card immediately after the initial diagnosis of patients, and all hospitals are obliged to report HFMD cases to the local Center for Disease Control and Prevention (CDC) within 24 h. Surveillance data included information on sociodemographic and clinical characteristics, such as age (<1 year/1–3 years/3–5 years/>5 years), sex (male/female), place of residence, month of onset, and disease severity (mild/severe/death). Cases with unknown addresses and no laboratory diagnoses were excluded. In addition, the demographic data of permanent residents were gathered from the Shanxi Provincial Bureau of Statistics.

### Specimen testing

Virological surveillance was carried out by the CDC in 11 prefecture-level cities in Shanxi, and all testing methods were conducted in accordance with relevant regulations and guidelines [28]. Throat swabs, anal swabs, and herpes test samples were collected from outpatients and inpatients at local hospitals. Real-time RT-PCR tests were performed to identify the enterovirus using ABI 7 500 fluorescence quantitative PCR instruments (ThermoFisher Scientific, Singapore) and enterovirus universal nucleic acid detection kits (DA AN GENE, Sun Yat-sen University, China). Without further serotype identification, the test results were divided into four groups: (1) enterovirus-negative, (2) EV71 positive, (3) CVA16 positive, and (4) positive with other enteroviruses. The exact names and proportions of the most frequently detected other enteroviruses [29] are listed in Supplementary Table S1. On the basis of the diagnostic criteria of HFMD, cases were classified as severe if they experienced cardiorespiratory failure, pulmonary oedema, and/or encephalitis; otherwise, they were classified as mild [30].

### Data analysis

#### Basic epidemiological and statistical analysis

Descriptive statistics, including demographic, seasonal, and aetiological distributions, were used to describe the epidemiological characteristics of HFMD. Chi-square tests were applied to compare differences in age, sex, and incidence rate of HFMD.

#### Spatial autocorrelation analysis

Spatial autocorrelation, divided into global and local autocorrelations, refers to the potential interdependence between the observed data of certain variables within the same distribution area. To understand the geographic characteristics of infections, we used the natural break method to divide the number of HFMD cases in 11 prefecture-level cities in Shanxi into four grades, draw spatial distribution maps with different colours, and then performed global and local spatial autocorrelation analysis using Moran's *I* index and 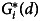
as research indicators. All analyses were conducted using ArcGIS (version 10.8, ESRI, Redlands, CA, USA).

#### Global spatial autocorrelation analysis

Our global spatial autocorrelation analysis used Moran's *I* index to reflect the degree of disease aggregation in the entire region. Moran's *I* index ranges from -1 to +1, indicating either a spatial positive correlation (aggregation distribution) or a spatial negative correlation (discrete mutually exclusion distribution) within the study area. The calculation formula is as follows:
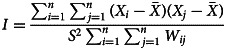
where *n* is the number of spatial units studied; *X*_*i*_ and *X*_*j*_ are the attribute values of regions *i* and *j*; 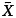 is the mean value of spatial units in the region; *S*^2^ is the variance; and *W*_*ij*_ is the spatial weight matrix, with adjacent values of 1 and non-adjacent values of 0.

#### Local spatial autocorrelation analysis

Our local spatial autocorrelation analysis reflected the spatial relationships of different element indicators in local areas. We used hotspot analysis to examine local spatial autocorrelation, which can distinguish the distribution characteristics of local spatial clusters using cold spots and hot spots. The model formula is as follows:
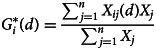


The higher the 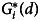 score (greater than 0), the closer the high-dimensional clustering of the target object attributes (forming hot spots); the lower the 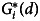 score (less than 0), the closer the low-dimensional clustering of the target object attributes (forming cold spots).

### Monthly incidence prediction

#### SARIMA model

The SARIMA model (*p*,*d*,*q*) × (*P*,*D*,*Q*)*_s_* is a common forecasting model for infectious diseases and can be used to fit seasonal time series. In the model, ‘S’ is the seasonal cycle, ‘AR’ is the autoregressive, ‘MA’ is the moving average, ‘*p*’ and ‘*P*’ are the number of autoregressive and seasonal autoregressive terms, respectively, ‘*d*’ and ‘*D*’ are the order of non-seasonal and seasonal differences, respectively, and ‘*q*’ and ‘*Q*’ are the number of moving average and seasonal moving average terms, respectively.

The prediction process of the SARIMA model is divided into four steps. The first step is stabilisation of the time series. The Augmented Dickey-Fuller (ADF) unit root test was used to judge whether the time series is stable. If not stationary, log transformations, differences, or seasonal differences are utilised to induce stationarity. The second step is model identification. The diagrams of the autocorrelation function (ACF) and partial correlation function (PACF) are plotted to preliminarily determine model patterns. The third step is model diagnosis. The optimal model was selected through parameter estimation and model testing. The normalised Bayesian information criterion (BIC) and coefficient of determination (*R*^2^) are used to compare the goodness-of-fit of models, and the Ljung-Box test is applied to determine whether the residual series is white noise. The fourth step is model prediction. The optimal combination of parameters is used to make predictions, and the errors between the predicted and actual values are calculated [22, 31]. The SARIMA model was developed by the R software (version 4.1.1, R Foundation for Statistical Computing, Vienna, Austria) with packages ‘forecast’ and ‘tseries’.

#### LSTM model

**Fig. 1.**
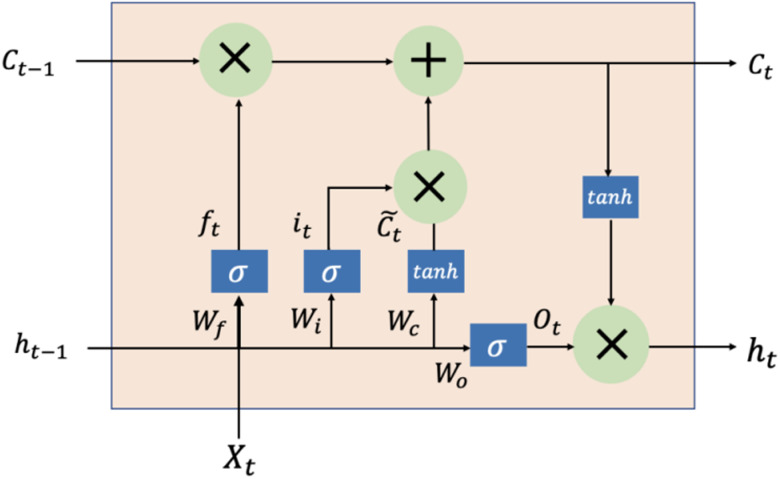
The structure of the LSTM model. *f*_*t*_, *i*_*t*_, *O*_*t*_ stand for the forget, input, and output gates, respectively; 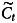 is the candidate memory cell state at time *t*; *C*_*t*_ is the cell state at time *t*; *h*_*t*_ is the hidden state at time *t*; *W* is the weight matrix; and *σ* is the sigmoid activation function.

The LSTM, proposed by Hochreiter and Schmidhuber in 1997, has been extensively used to solve time-series problems with long-term dependencies [32]. The three gates (input, output and forget) and cell state are the core concepts of the LSTM. The LSTM is special type of RNN that can overcome the defect of RNN sensitive to short-term inputs by introducing gate structures and a well-defined cell state [33]. These gates can determine what information should be added and stored, or forgotten and removed during training. Figure 1 displays the structure of the LSTM model, and the following equations are used to define it:
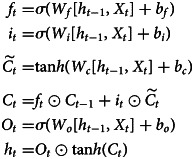
where *f*_*t*_, *i*_*t*_, *O*_*t*_ stand for the forget, input, and output gates, respectively; 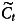 is the candidate memory cell state at time *t*; *C*_*t*_ is the cell state at time *t*; *h*_*t*_ is the hidden state at time *t*; *W* is the weight matrix; *b* is the bias term; and *σ* is the sigmoid activation function.

We utilised Python software (version 3.7.1, Python Software Foundation, Python Language Reference) to construct the LSTM model with packages ‘tensorflow’ and ‘keras.’ To shorten the training time of the network and accelerate the gradient descent, the source data were processed by adopting the maximum and minimum normalisation method to restrict the values between 0 and 1. Additionally, the data of the last 12 months were split as the test set in the prediction, while the rest were split for the training set. We then used the different time steps, hidden neurons, and optimisers to choose the optimal model depended on the minimum root mean square error (RMSE) of the test set. The best set of hyperparameters was selected to produce out-of-sample predictions, and the predicted values were normalised inversely.

#### Measuring for accuracy

We limited the data analysis from January 2017 to August 2021 in order to develop prediction models, using the subsequent 12 months for testing. The mean absolute error (MAE), mean absolute percentage error (MAPE), and RMSE were used to evaluate the predictive performance and accuracy of the established models. The MAE, MAPE, and RMSE are defined as follows:
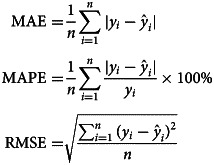
where *y*_*i*_ and 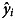 represent the actual and predicted values, respectively; *n* is the number of simulations and predictions in the models used.

## Results

### Epidemiological characteristics of HFMD

#### Demographic distribution of HFMD

**Fig. 2.**
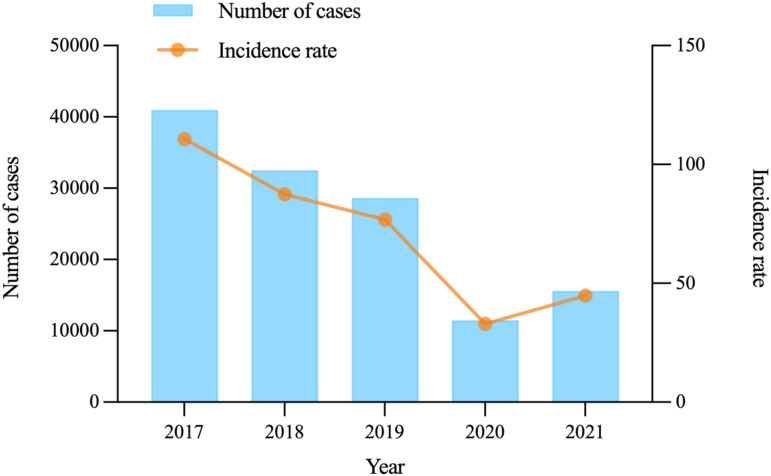
Number of HFMD cases and annual incidence rates in Shanxi Province from 2017–2021.

In Shanxi, 129 288 HFMD cases were reported to the surveillance system from 2017 to 2021. Of these, 554 cases were diagnosed with severe cases and there were no fatal case. The incidence of reported HFMD cases showed a significant downward trend from 2017–2020 (*χ^2^* = 13 689.397, *P* < 0.001); however, the incidence increased in 2021, with an annual average incidence of 71.34/100 000 in the entire population. The incidence rates of HFMD showed broad age-specific variation (*χ^2^* = 465.937, *P* < 0.001). The proportion of patients with HFMD aged <5 years accounted for 86.78% of the total number of cases. Furthermore, the most severe cases were in patients aged <3 years, accounting for 78.16%. During the five years, higher HFMD incidence rates were noted in male patients (*χ^2^* = 28.608, *P* < 0.001), and the male-to-female relative risk (*RR*) was 1.316 (Table 1 and Figure 2).

**Table 1.** Demographic distribution of HFMD in Shanxi Province from 2017–2021

Year	Number of cases	Incidence rate (/100 000)	Number of severe cases	Proportion of mild cases (%)	Age *n* (%)				Gender *n* (%)	
					<1 year	1–3 years	3–5 years	>5 years	Male	Female
2017	40 994	110.72	479	98.83	2 070 (5.05)	25 719 (62.74)	8 945 (21.82)	4 260 (10.39)	23 990 (58.52)	17 004 (41.48)
2018	32 526	87.47	42	99.87	1 245 (3.83)	19 779 (60.81)	7 093 (21.81)	4 409 (13.56)	18 888 (58.07)	13 638 (41.93)
2019	28 655	76.84	22	99.92	745 (2.60)	15 723 (54.87)	7 529 (26.27)	4 658 (16.26)	16 339 (57.02)	12 316 (42.98)
2020	11 495	32.92	0	100.00	682 (5.93)	7 227 (62.87)	2 288 (19.90)	1 298 (11.29)	6 721 (58.47)	4 774 (43.74)
2021	15 618	44.87	11	99.93	702 (4.49)	8 348 (53.45)	4 099 (26.25)	2 469 (15.81)	8 835 (56.57)	6 783 (43.43)

#### Seasonal distribution of HFMD

**Fig. 3.**
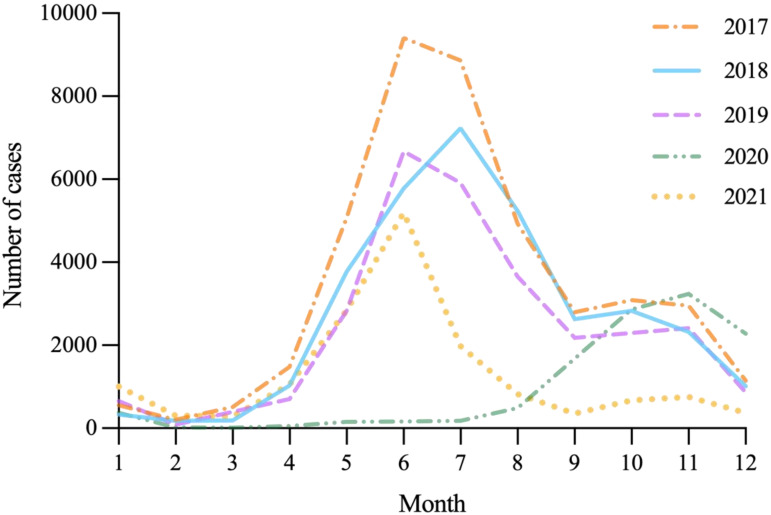
Seasonal distribution of HFMD in Shanxi Province from 2017–2021.

HFMD was epidemic throughout the year in Shanxi, with a single peak in November 2020. In the other four years, annual epidemic waves were observed, with major peaks in early summer (June and July), followed by secondary peaks in autumn (October and November). Moreover, with the exception of 2020, the summer and autumn peaks were lower in height than in previous years (Figure 3).

#### Aetiologic distribution of HFMD

From 2017–2021, the successive annual positive rates of HFMD enterovirus infection in Shanxi were 68.35%, 59.43%, 63.32%, 64.78%, and 71.08%, all exceeding or close to 60.00%. Of these, 14 049 (16.72%), 23 586 (28.06%), and 52 643 (62.64%) cases were associated with EV71, CVA16, and other enteroviruses (including 22 cases positive for both EV71 and CVA16, 2 272 cases positive for both EV71 and other enteroviruses, 3 951 cases positive for both CVA16 and other enteroviruses, and 11 cases positive for EV71, CVA16, and other enteroviruses), respectively. With the exception of 2019, when CVA16 was the primary attacking enterovirus, other enteroviruses were the predominant causative agents of HFMD, with percentages increasing from 51.75% to 90.37%. In addition, fewer cases of infection with two enteroviruses during the study period were noted, with only 11 cases simultaneously having multiple enteroviruses (Table 2).

**Table 2.** Aetiologic distribution of HFMD in Shanxi Province from 2017–2021

Year	Positive cases *n* (%)	Enterovirus serotypes *n* (%)						
		EV71	CVA16	Other enteroviruses	EV71 & CVA16	EV71 & Other enteroviruses	CVA16 & Other enteroviruses	EV71 & CVA16 & Other enteroviruses
2017	28 019 (68.35)	12 626 (45.06)	3 939 (14.06)	14 500 (51.75)	22 (0.08)	2 118 (7.56)	917 (3.27)	11 (0.04)
2018	19 331 (59.43)	912 (4.72)	6 698 (34.65)	12 865 (66.55)	0 (0)	110 (0.57)	1 034 (5.35)	0 (0)
2019	18 145 (63.32)	187 (1.03)	11 150 (61.45)	8 623 (47.52)	0 (0)	44 (0.24)	1 771 (9.76)	0 (0)
2020	7 447 (64.78)	11 (0.15)	858 (11.52)	6 622 (88.92)	0 (0)	0 (0)	44 (0.59)	0 (0)
2021	11 102 (71.08)	313 (2.82)	941 (8.48)	10 033 (90.37)	0 (0)	0 (0)	185 (1.67)	0 (0)

### Spatial autocorrelation analysis

#### Spatial distribution of HFMD

**Fig. 4.**
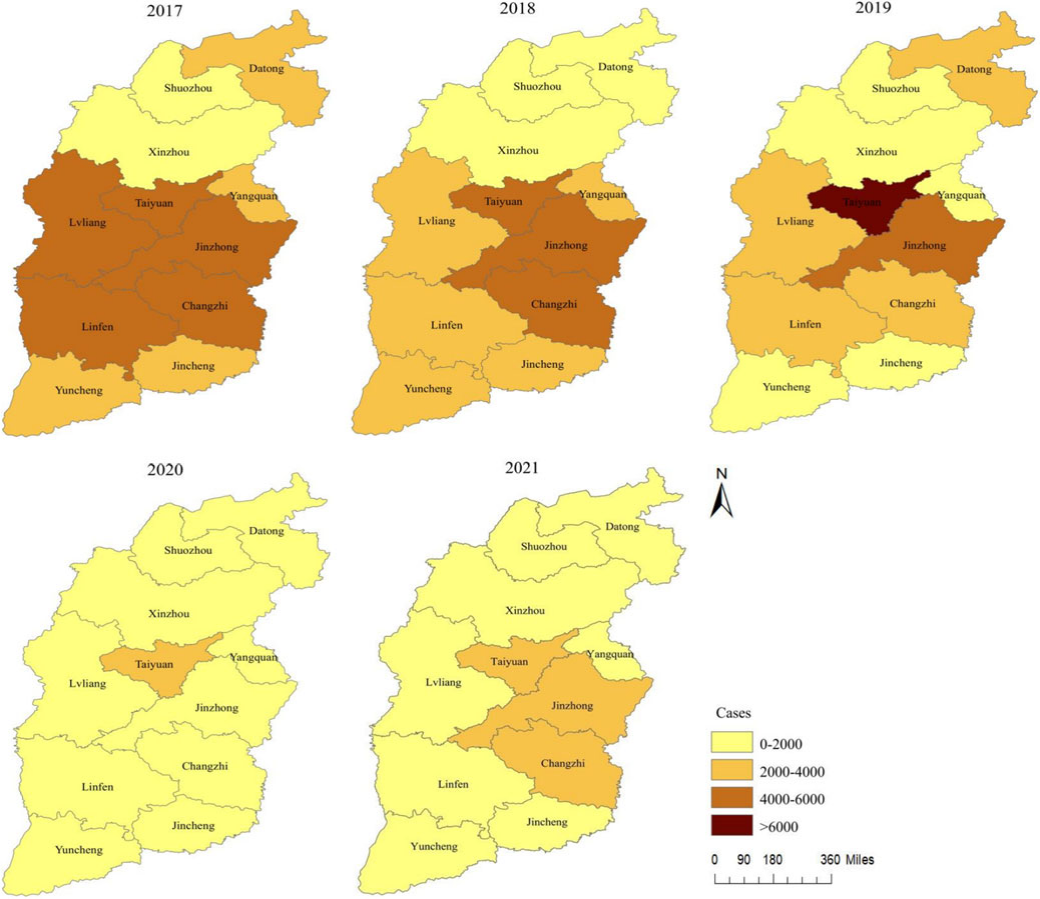
Spatial distribution of HFMD in Shanxi Province from 2017–2021.

There are 11 prefecture-level cities in the province of Shanxi, and the number of HFMD cases varied substantially among these cities (Figure 4). From 2017 to 2021, the number of cases ranged from 0 (Shuozhou in 2020) to 6 600 (Taiyuan in 2019), and although the epidemic intensity differed, trends were similar. From 2017 to 2021, areas with a large number of HFMD cases were primarily concentrated in central Shanxi, such as Taiyuan, whereas the number of HFMD cases in northern areas, such as Xinzhou, was relatively small. The regional, demographic, economic, and meteorological profiles of the 11 prefecture-level cities are displayed in Table 3.

**Table 3.** Regional, demographic, economic, and meteorological profiles of the 11 prefecture-level cities in Shanxi Province

City	Regional profile	Demographic profile			Economic profile	Meteorological profile		
	Location	Minimum	Maximum	Median	GDP per capita	Average latitude	Average altitude (m)	Average temperature (°C)
Taiyuan	Central Shanxi	4 984 000	5 391 000	5 232 000	95 600	37°87′	791	8.1–11
Datong	Northern Shanxi	3 099 000	3 161 000	3 128 000	54 400	40°08′	1 052	6.5–8.6
Yangquan	Central-eastern Shanxi	1 311 000	1 326 000	1 321 000	69 700	37°85′	657	8–12
Changzhi	Southeastern Shanxi	3 152 000	3 213 000	3 186 000	73 000	36°20′	930	6–17
Jincheng	Southeastern Shanxi	2 189 000	2 202 000	2 195 000	87 300	35°50′	711	10.2–12
Shuozhou	Northern Shanxi	1 590 000	1 632 000	1 604 000	89 300	39°33′	1 094	3.6–7.3
Jinzhong	Central Shanxi	3 348 000	3 390 000	3 375 000	54 500	37°68′	828	5–19
Yuncheng	Southwestern Shanxi	4 733 000	4 855 000	4 789 000	43 200	35°02′	374	10–21
Xinzhou	Northern Shanxi	2 663 000	2 811 000	2 720 000	50 300	38°42′	789	4.3–9.2
Linfen	Southwestern Shanxi	3 912 000	4 076 000	4 000 000	48 400	36°08′	459	10–21
Lvliang	Central-western Shanxi	3 375 000	3 497 000	3 418 000	61 200	37°52′	945	6–18

#### Global spatial autocorrelation analysis

The successive annual global Moran's *I* index values of HFMD in Shanxi from 2017 to 2021 were 0.508, 0.502, 0.025, -0.160, and 0.053. In 2017 and 2018, the *P*-values were less than 0.05, indicating global autocorrelation. As the Moran's *I* index values in 2017 and 2018 were greater than 0, the spatial clusters of HFMD manifested a certain global spatial positive correlation. Conversely, the *P*-values of the other years were greater than 0.05, indicating no statistical significance.

#### Local spatial autocorrelation analysis

**Fig. 5.**
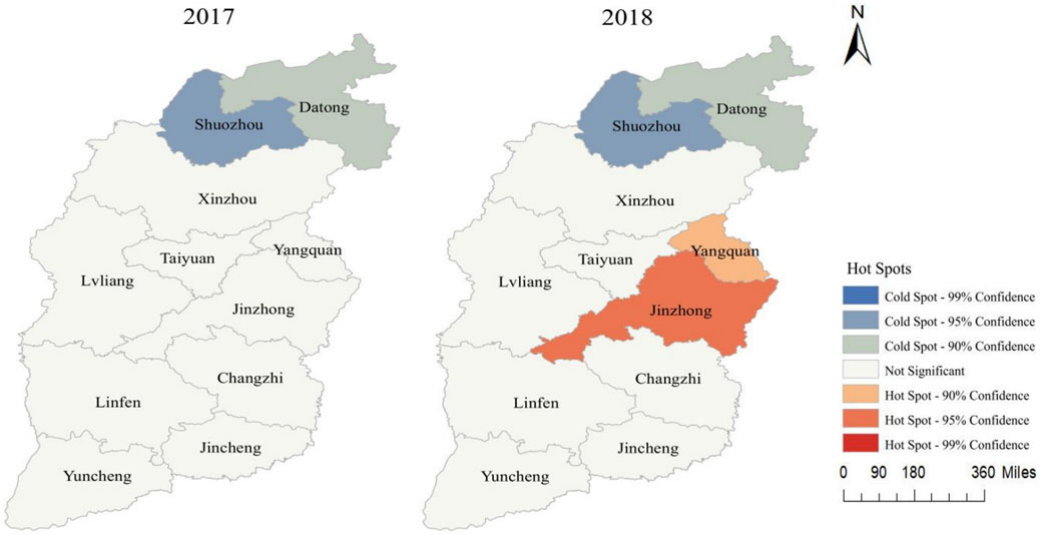
Results of local spatial autocorrelation analysis in Shanxi Province from 2017–2018.

Hotspot analysis divided the spatial distribution of HFMD cases into seven levels: (1) high hot spots, (2) hot spots, (3) secondary hot spots, (4) high cold spots, (5) cold spots, (6) secondary cold spots, and (7) no significant spots. As shown in Figure 5, from 2017 to 2018, the cold spots and secondary cold spots in Shanxi were concentrated in Shuozhou and Datong. In 2018, contrastingly, the hot spots and secondary hot spots were concentrated in Jinzhong and Yangquan.

### Monthly incidence prediction

#### SARIMA model

**Fig. 6.**
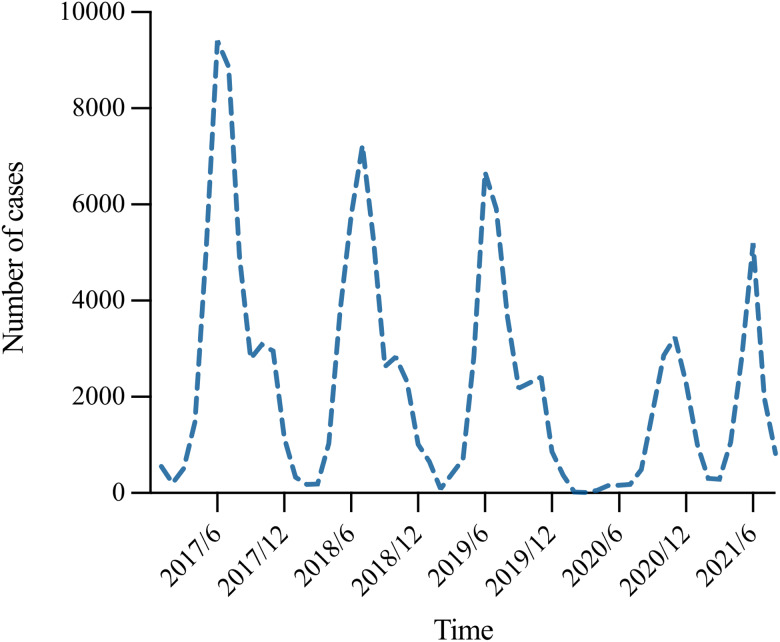
Sequence diagram of HFMD cases in Shanxi Province from January 2017 to August 2021.

**Fig. 7.**
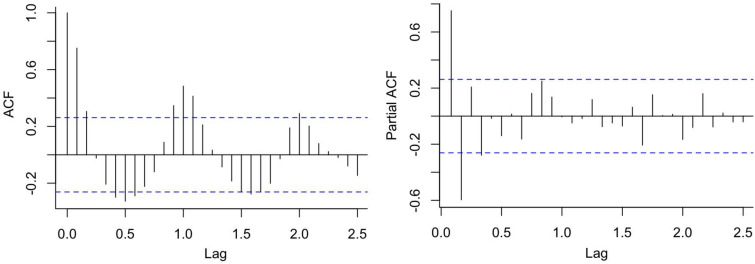
ACF and PACF of the source data.

**Fig. 8.**
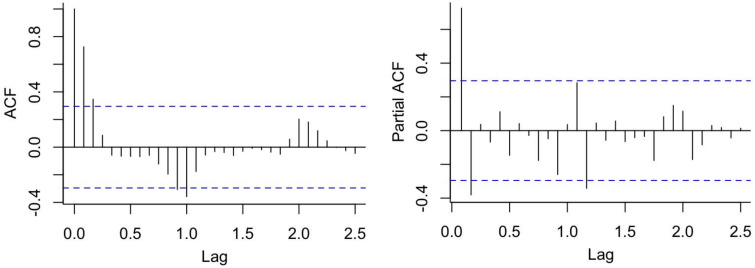
ACF and PACF after first-order seasonal difference.

According to the sequence diagram, the data presented an obvious seasonal trend, requiring the use of first-order seasonal difference (Figure 6). The seasonal decomposition diagram is displayed in Supplementary Figure S1. After the first-order seasonal difference, the time sequence was stationary (ADF = −4.936, *P* < 0.01). Figure 7 shows the ACF and PACF of the source data, and Figure 8 shows the ACF and PACF after the first-order seasonal difference. Based on the comparative results of the various goodness-of-fit tests, our study identified the optimal SARIMA(2,0,0)(1,1,0)_12_ model, which had the lowest BIC (14.100) and the highest *R*^2^ (0.901). The Q-Q plot shows that the residuals were essentially normally distributed (Supplementary Figure S2). The Ljung-Box test demonstrated that the residuals were white noise (*P_Ljung-Box_* = 0.988), verifying that the fitted data was completely summarised. Table 4 displays the parameter estimation for the SARIMA(2,0,0)(1,1,0)_12_ model, which were found to be statistically significant.

**Table 4.** Parameter estimation for SARIMA(2,0,0)(1,1,0)_12_ model

Variable	*B*	*SE*	*t*	*P*
AR(1)	1.183	0.130	9.110	<0.001
AR(2)	-0.559	0.131	-4.273	<0.001
SAR(1)	-0.703	0.127	-5.555	<0.001

#### LSTM model

In the LSTM network, the time-slice steps of the data sample were set to three/six, indicating that we used the data of the previous three/six months to predict the incidence of the next month. A neural network structure with one hidden layer was adopted with neuron options of 16/32/64/128, and the alternative optimisers were Adaptive Moment Estimation (Adam) and Stochastic Gradient Descent (SGD). In addition, a fully connected layer was created with an output dimension of one. The model used a training wheel designed for 200 rounds with a batch size of one, and the mean square error (MSE) was chosen as the loss function. To avoid overfitting, the dropout method was applied to the non-circular part of the hidden layer to randomly deactivate neurons with a dropout value of 0.1. The ten alternative LSTM models are listed in Supplementary Table S2. Finally, we confirmed that the preferred model with six time steps, one hidden layer involving 128 hidden neurons, and the Adam optimiser had the lowest RMSE for the test set (RMSE = 461.96), compared with models using other combinations of hyperparameters.

#### Model comparison

**Fig. 9.**
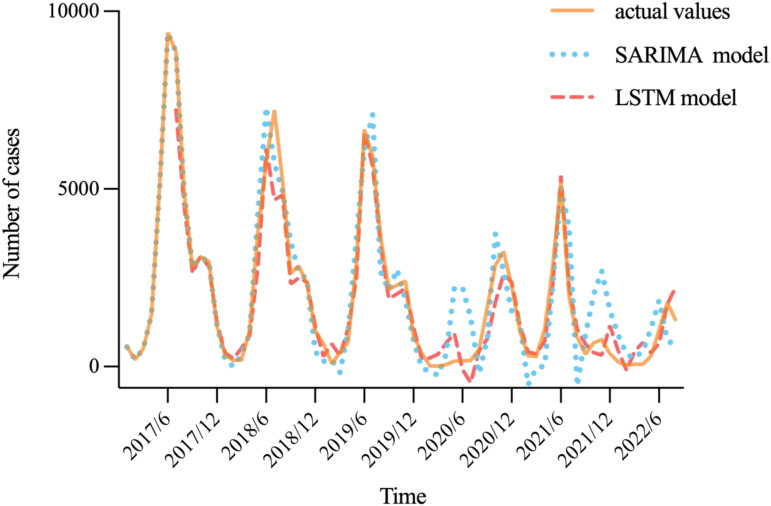
Prediction diagram of SARIMA(2,0,0)(1,1,0)_12_ model and LSTM model.

The simulated and predicted performances of the SARIMA and LSTM models were compared using multiple statistical indicators. Figure 9 shows that the simulation and prediction trends of HFMD using both models were relatively consistent with the actual situation, verifying that the established models were reliable in assessing the epidemic trend. Among the two techniques, the LSTM model performed well in the prospective forecasting of HFMD prevalence over the following 12 months, with a lower MAE (386.58 *vs.* 838.25), MAPE (2.25 *vs.* 3.08), and RMSE (461.96 *vs.* 963.13). This indicated that the LSTM model was more appropriate than the SARIMA model in predicting the monthly incidence of HFMD (Table 5).

**Table 5.** Comparison of the predicted and actual values of the SARIMA(2,0,0)(1,1,0)_12_ model and LSTM model

Month/Year	Actual values	SARIMA model	LSTM model
September 2021	353	887	645
October 2021	667	2 019	398
November 2021	753	2 691	319
December 2021	368	1 588	1 153
January 2022	125	879	442
February 2022	38	351	-105
March 2022	64	239	432
April 2022	64	444	680
May 2022	295	1 064	387
June 2022	1 026	1 856	654
July 2022	1 799	755	1 750
August 2022	1 320	570	2 222
Simulated performance	MAE	518.36	383.80
	MAPE	1.60	1.55
	RMSE	752.50	578.00
Predicted performance	MAE	838.25	386.58
	MAPE	3.08	2.25
	RMSE	963.13	461.96
Simulated performance reduced percentage (%) LSTM *vs.* SARIMA	MAE	25.96%	
	MAPE	3.13%	
	RMSE	23.19%	
Predicted performance reduced percentage (%) LSTM *vs.* SARIMA	MAE	53.88%	
	MAPE	26.95%	
	RMSE	52.04%	

## Discussion

We studied the data of HFMD in Shanxi from 2017 to 2021 which contained 129 288 HFMD cases. The dataset used in our study was the most comprehensive dataset describing the latest characteristics of HFMD in Shanxi. This study confirmed that the prevalence of HFMD in this province had significant demographic, seasonal, aetiologic, and spatial characteristics, and that the LSTM model was a useful technology for building an early warning system for HFMD. Although the epidemic tendency was similar with the findings reported in the vast majority of northern China, some differences were observed in a few areas [19, 34]. For example, though with similar demographic and seasonal distributions to Shanxi Province, EV71, rather than other enteroviruses, has been the predominant enterovirus serotype in Xi'an, Shaanxi Province since 2011.

From 2017 to 2020, the incidence of HFMD in Shanxi showed a significant trend of decrease, and the overwhelming majority of patients experienced only mild symptoms, indicating that the prevention and control measures in place for HFMD had achieved some success. Compared to the world, Shanxi had a relatively low incidence rate [35]. However, the incidence appeared to rebound in 2021. In the face of a severe epidemic across the country [36], every effort to reduce the spread of HFMD is vital.

By summarising the demographic data over the five-year period, we found that the incidence of HFMD was higher in males than in females. This may be due to males being naturally more active and having a wider range of activities. These factors greatly increase the chances of exposure to the virus and easily cause cross infection [37]. In addition, the majority of patient with HFMD in Shanxi were young children aged <5 years, with those aged <3 years most affected by severe HFMD. This may be due to low resistance in children in this age group as well as a lack of basic knowledge for HFMD prevention among parents [38]. Therefore, improving vaccination rates for HFMD among young children and increasing HFMD health knowledge among parents is critical.

With the exception of 2020, the largest number of outbreaks of HFMD in Shanxi primarily occurred in the months of June and July, followed by October and November. Temperature and humidity influence the enterovirus activity. A systematic review found a statistically significant positive relationship between HFMD cases and both temperature and humidity [39]. The increase in temperature and humidity in summer accelerates the growth and reproduction of the enterovirus, which is conducive to the spread of HFMD. However, the seasonal distribution of HFMD in 2020 showed a ‘single-peak’ pattern, with only one outbreak in November. This situation was speculated to be related to the COVID-19 pandemic in the first half of 2020. The government took comprehensive intervention measures, including strict restrictions on the movement of people and short-term closing of kindergarten, thereby cutting off the transmission route of COVID-19 and HFMD. These results suggest that intervention efforts should be vigorously pursued prior to expected HFMD infection peaks. Furthermore, according to the average growth from the previous year 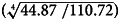, the epidemics were successively smaller, indicating that HFMD may have gradually been controlled.

In terms of transmissibility, EV71 can cause widespread epidemics of HFMD, and in terms of pathogenicity, EV71 is consistently the predominant pathogen in severe cases and deaths, with 74% of severe cases and 93% of deaths associated with EV71 [40]. CVA16 has a broad spectrum of pathogenicity and can cause a variety of diseases such as herpetic angina, myocarditis, and aseptic meningitis, but the clinical symptoms are relatively mild [41]. The incidence of HFMD caused by other enteroviruses has increased significantly in recent years, with CVA6 causing a more extensive rash than CVA16 and EV71. In a Japanese study, CVA6 and CVA10 were shown to be less virulent than EV71 during the HFMD epidemic [42]. In the present study, other enteroviruses were the predominant causative agents of HFMD in Shanxi during the study period, with the exception of 2019, when CVA16 was the primary attacking enterovirus. This is contrary to the conclusion that EV71 is more transmissible, virulent, and pathogenic than CVA16 and other enteroviruses [43]. We conjectured that this may be associated with the reduction in the number of susceptible people caused by large-scale EV71 epidemics in previous years. At present, people may have established a certain degree of immune barrier against EV71, but may be more sensitive to CVA16 and other enteroviruses. Moreover, the incidence of HFMD has decreased significantly with the launch of the inactivated monovalent EV71 vaccine. However, while this vaccine may reduce the occurrence of EV71-associated HFMD, it is not effective against other aetiologies. Enterovirus serotype replacement highlighted the importance of laboratory-based surveillance and suggested that a focus on CVA16 and other enteroviruses by the CDC may be needed.

This study also indicated that, from 2017–2021, the areas with large numbers of HFMD cases were primarily concentrated in the central part of Shanxi, such as the provincial capital of Taiyuan and its neighbour cities. In contrast, the number of HFMD cases in northern areas, such as Xinzhou, was relatively small. These findings may be mainly related to high population densities, large floating populations, relatively developed economies, and relatively high temperatures in the central regions. In 2017 and 2018, the spatial clusters of HFMD manifested a certain global spatial positive correlation, showing that the areas with higher incidence were adjacent to each other and the areas with lower incidence were also adjacent to each other. The global Moran's *I* index cannot accurately orient the spatial cluster location of the disease; however, in practice, it is often necessary to determine which areas are high-incidence clusters (hot spots) and which areas are low-incidence clusters (cold spots). The results of hotspot analysis showed that cold and secondary cold spots were concentrated in Shuozhou and Datong in 2017 and 2018, whereas hot and secondary hot spots were concentrated in Jinzhong and Yangquan only in 2018. After 2018, in order to prevent the emergence of aggregated epidemics and severe cases, the health and family planning departments of 11 cities in Shanxi Province worked in collaboration with the education sectors, focusing on schools and childcare institutions to vigorously carry out prevention and treatment of HFMD, while strengthening publicity and education for key populations and providing standardised vaccination services. The cases of HFMD in 11 cities showed a certain ‘uniform distribution’ characteristic, so no cold spots or hot spots appeared.

At present, ARIMA model has been widely used to simulate and forecast the epidemic tendency of infectious diseases and has achieved satisfactory effects [26, 44]. In this work, we established a multiplicative ARIMA model due to the seasonal variations and annual periodicity of HFMD in Shanxi. Based on the comparative results of the various goodness-of-fit tests, the SARIMA(2,0,0)(1,1,0)_12_ model was optimal, with the lowest BIC and highest *R*^2^, and could reliably forecast the number of HFMD patients. However, the SARIMA model may have difficulties capturing the nonlinear characteristics of infectious disease data [25]. We also used the LSTM network for prediction due to its flexible capacity to determine what to add or remove during the training as well as it having the ability to effectively address the nonlinear dynamics and long-term temporal dependencies present in sequential data [23]. Given that LSTM model has performed well in predicting the incidence of other infectious diseases with similar epidemiological mechanisms to HFMD, the application of LSTM technique to HFMD in this study is considered practical and feasible. A neural network structure of six time steps, 128 hidden neurons, and the Adam optimiser were found to provide optimal predictive performance with an RMSE of 461.96. Our results implied that the MAE, MAPE, and RMSE of the LSTM model were lower than those of the SARIMA model in both the training and test sets. The LSTM method may reduce the values of the three statistical indicators mentioned above in the training set by 25.96%, 3.13%, and 23.19%, respectively, and decrease the corresponding values in the test set by 53.88%, 26.95%, and 52.04%, respectively, compared with the SARIMA model. This indicated that the LSTM model had better forecast accuracy of HFMD for time series with periodic characteristics and may provide a clearer perspective of popular trends. The SARIMA model is constructed on the premise of differencing the original series to eliminate seasonal trends, which could potentially lead to under-utilisation of information and result in forecasting errors, whereas the LSTM network has no requirement for the data itself to be stable. Therefore, we inferred that the LSTM method should be emphasised when predicting the prevalence of infectious diseases.

This study had several limitations. First, only EV71 and CVA16 serotypes were detected by the local CDC, and other specific serotypes, such as CVA6 and CVA10, were not tested. Second, the incidence of HFMD is complex and changeable, and may be affected by climatic factors, social development, and population immunity levels [45, 46]. The influence of these exogenous variables was not considered in this study when constructing the prediction models. Third, prediction is a continuous dynamic process, and its results are sensitive to the choice of parameters for each module of the model. Therefore, the model should be updated in practice according to different conditions and time periods to ensure its strength in predictive performance. Finally, both the SARIMA model and the LSTM model we constructed were driven by the surveillance data of HFMD under real-world conditions, so it was difficult to take into account the impact of the COVID-19 pandemic in the prediction. Efforts must be made to comprehensively identify the serotypes of enteroviruses, explore an optimal forecasting model in combination with exogenous variables, and quantitatively measure the impact of anti-COVID-19 nonpharmaceutical interventions in predicting the number of HFMD cases.

## Conclusion

Our study was the first to explore the three aspects of HFMD: epidemiological characteristics, spatial clusters, and monthly incidence prediction, fully investigating the fundamental characteristics of the disease. We found that the incidence of HFMD in Shanxi has generally declined, and that children younger than five years of age, particularly boys, were the main group affected. Seasonal outbreaks occurred in summer and autumn, and other enteroviruses were the predominant causative agents of HFMD. Additionally, the central regions of Shanxi were hot spots for HFMD incidence. The LSTM model proposed in this study reliably forecasted the monthly incidence of HFMD, which may provide technical support in constructing an HFMD early warning system. These findings may help policymakers allocate health resources reasonably and preemptively prepare for possible epidemics of HFMD in Shanxi and other parts of northern China.

## Data Availability

The data that support the findings of this study are available from the corresponding author (Hongmei Yu), upon reasonable request.

## Supplementary material

For supplementary material accompanying this paper visit http://doi.org/10.1017/S0950268823000389. click here to view supplementary material
