# Supplementary material for: Epidemiological characteristics, spatial clusters and monthly incidence prediction of hand, foot and mouth disease from 2017 to 2022 in Shanxi Province, China
Source: Epidemiol Infect. 2023 Mar 14;151:e54. doi: 10.1017/S0950268823000389 (PMC10126901; doi:10.1017/S0950268823000389)
Supplement: Supplementary file 1 [file S0950268823000389sup001.docx]

*Epidemiology and Infection*, **Epidemiological characteristics, spatial clusters, and monthly incidence prediction of hand, foot, and mouth disease from 2017 to 2022 in Shanxi Province, China,** Yifei Ma, Shujun Xu, Ali Dong, Jianhua An, Yao Qin, Hui Yang, Hongmei Yu

Supplementary Material

**Supplementary Figure S1**
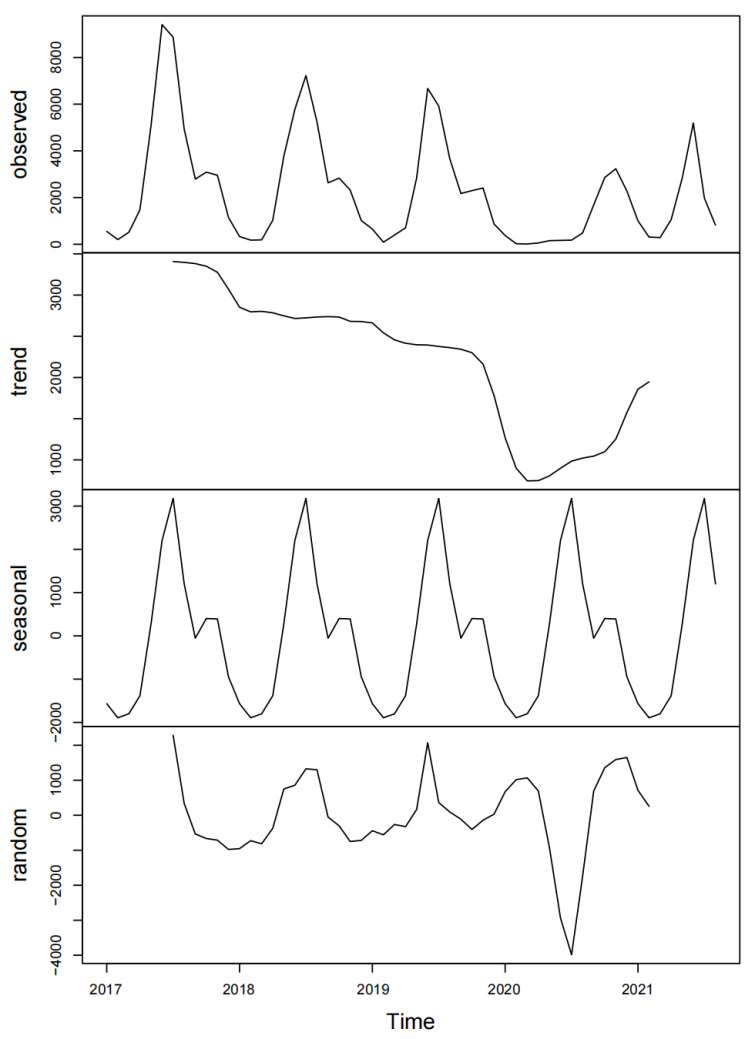
 Seasonal decomposition diagram of HFMD cases in Shanxi Province from January 2017 to August 2021

**Supplementary Figure S2** Q-Q
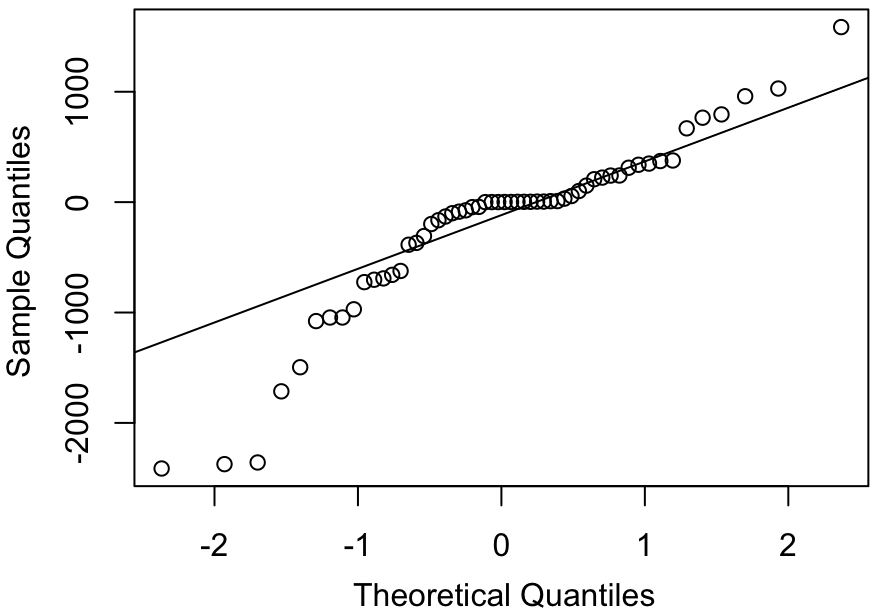
 plot of the SARIMA(2,0,0)(1,1,0)_12_ model residuals

**Supplementary Table S1** Exact names and proportions of the most frequently detected other enteroviruses

| Names | Proportions |
| --- | --- |
| CVA6 | 41.47% |
| CVA10 | 26.66% |
| CVA2 | 2.54% |
| CVA4 | 1.55% |
| Echo6 | 1.16% |
| CVB1 | 1.13% |
| CVA5 | <1% |
| CVA12 | <1% |
| CVA14 | <1% |
| CVB3 | <1% |
| CVB4 | <1% |
| CVB5 | <1% |
| Echo3 | <1% |
| Echo7 | <1% |
| Echo8 | <1% |
| Echo9 | <1% |
| Echo11 | <1% |
| Echo16 | <1% |
| Echo18 | <1% |

**Supplementary Table S2** Comparison of the ten alternative LSTM models

| Model | Time Steps | Hidden neurons | Optimizer | RMSE |
| --- | --- | --- | --- | --- |
| **1** | **six** | **128** | **Adam** | **461.96** |
| 2 | six | 16 | Adam | 726.18 |
| 3 | six | 32 | Adam | 593.88 |
| 4 | six | 64 | Adam | 523.07 |
| 5 | six | 128 | SGD | 761.85 |
| 6 | three | 128 | Adam | 481.55 |
| 7 | three | 16 | Adam | 782.69 |
| 8 | three | 32 | Adam | 627.55 |
| 9 | three | 64 | Adam | 624.55 |
| 10 | three | 128 | SGD | 1043.49 |
